# Combustion Behavior and Mechanism of Ti14 Titanium Alloy

**DOI:** 10.3390/ma13030682

**Published:** 2020-02-03

**Authors:** Lei Shao, Guoliang Xie, Hongying Li, Wanran Lu, Xiao Liu, Jiabin Yu, Jinfeng Huang

**Affiliations:** State Key Laboratory for Advanced Metals and Materials, University of Science and Technology Beijing, No. 30, Xueyuan Road, Beijing 100083, China; b20170442@xs.ustb.edu.cn (L.S.); delialhy@163.com (H.L.); lwrygmljq@163.com (W.L.); dxhzyq_fighting@163.com (X.L.); 13161879524@163.com (J.Y.); ustbjinfeng_huang@163.com (J.H.)

**Keywords:** titanium fire, combustion mechanism, burning velocity, elements enrichment

## Abstract

The combustion behavior and mechanism of Ti14 titanium alloy are studied by promoted ignition combustion tests at different oxygen pressures in this paper. The burning velocity increases at higher oxygen pressures and also increases with longer burning times instead of a constant at the same pressure. The Cu atoms are found enriched in two zones—i.e., the heat affected zone and melting zone during the combustion process—which can prevent the diffusion process of oxygen atoms. The different combustion behavior of Ti14 and Ti-Cr-V alloys is basically controlled by the characteristics of phase structures and chemical reactions.

## 1. Introduction

Titanium alloys have broad applications in aviation industry because of the excellent properties such as high strength, low density, and high corrosion resistance [1,2]. However, they can be ignited by high speed friction and particle impact under the conditions of high pressure and temperature due to the low thermal coefficient and high combustion heat, known as a “titanium fire” accident [3]. The applications of titanium alloys are limited by the “titanium fire” accident since the burning velocity of titanium alloys is so fast, needing only 4–20 s, that it can hardly be terminated once the combustion reaction starts.

To avoid this problem, many researchers are developing burn resistant titanium alloys. There are two typical types of burn resistant titanium alloys, one type is Ti-Cr-V system alloys and another is Ti-Cu system alloys. The burn resistant mechanism of Ti-Cr-V alloys is that adding some V and Cr (above 13 wt %) to Ti matrix, the burning product V_2_O_5_ is volatile and takes away a great deal of heat by volatilization process during combustion. Furthermore, the element Cr can form a dense and continuous oxide layer Cr_2_O_3_ in the combustion process and it can prevent the Ti matrix from oxidizing. Therefore, the Ti-Cr-V system alloys avoid combustion to some extent [4,5,6,7]. The Alloy C, Alloy C^+^, and Ti40 are Ti-Cr-V system alloys [8,9,10]. Different from the Ti-Cr-V alloys, the Ti-Cu system burn resistant alloys are based on the principle of friction. The copper shows wonderful thermal conductivity and it can transfer heat rapidly from friction to avoid the local heat concentration which makes it difficult to reach the ignition point [11,12,13]. The friction conditions are improved by changing dry friction into wet friction with liquid lubrication due to the melting of Ti_2_Cu phase, leading to the friction heat decreases sharply [14,15,16]. Therefore, the addition of Cu into titanium alloy is reported to improve the burn resistance. Several Ti-Cu system alloys have been developed, such as BTT-1, BTT-3, and Ti14 [17,18,19,20].

Ti-Cu system alloy is one of the most promising burn resistant alloys because of its lower costs and density than Ti-Cr-V alloys and extremely excellent workability. Several works have been conducted to study the combustion behavior of Ti-Cu alloy [14,17,18], whereas the burning velocity and combustion mechanism of Ti-Cu alloys is still unclear, especially the elements diffusion and phase transitions during combustion. Ti14 alloy is developed as a representative Ti-Cu system alloy for the applications in aircraft industries. In this paper, the combustion behavior and mechanism of Ti14 alloy are studied by the promoted ignition combustion (PIC) tests [21]. The non-isothermal oxidation, combustion velocity, and microstructure after combustion are studied, and the combustion mechanism of Ti14 alloy is discussed.

## 2. Materials and Methods

### 2.1. Material Preparation

Alloy ingots Ti14 with a nominal composition of Ti-1Al-13Cu-0.2Si was fabricated by vacuum arc melting. Those ingots were melted five times to ensure the chemical homogeneity, then heat treated at 810 °C for 0.5 h followed by water quenching to room temperature. After that, the ingots were hot rolled into 5 mm thick plate at 810 °C, and finally cut into rods with the dimension of Φ3.2 × 70 mm. Table 1 presents an analysis of the chemical composition of the experimental alloys.

### 2.2. Experimental Methods

**Figure 1:**
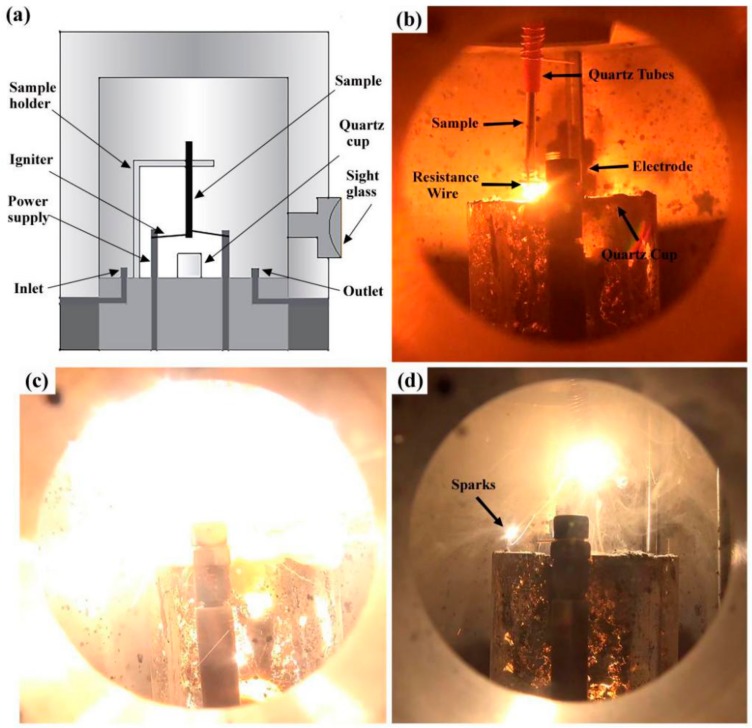
Illustration of promoted ignition combustion equipment (**a**), and the combustion process of titanium alloys (**b**) to (**d**), (**b**) thermo oxidation, (**c**) ignition, (**d**) flame expansion, respectively.

The PIC tests were carried which were wildly used in oxygen enriched atmosphere and the test procedures were described in detail in Refs. [22] and [23]. The quartz tubes with the internal diameter of 3.2 mm and the length of 20 mm were put on the samples at the position corresponding to the sample lengths of 10, 20, 30, and 40 mm, for the determination of burning rate in different stages. Argon gas is simultaneous pumped into the chamber to quench the sample. The burning time was measured of different combustion lengths to calculate the burning velocity. The equipment of PIC test and the combustion process were shown in Figure 1. The PIC tests were carried out at the oxygen pressure from 0.2 MPa to 0.5 MPa and each test was repeated three times to ensure the reliability of the experimental date.

### 2.3. Microstructural Characterizations

The samples after combustion were cut long longitudinal section in half, polished and etched in an solution of HF:HNO_3_:H_2_O = 1:3:6 for microstructure observation. The phase formation of combustion product was determined by X-ray diffraction (XRD) using a Huber-2 goniometer with a Cu target (TTR3, Rigaku, Tokyo, Japan). The microstructure characterization of combustion areas was conducted by optical microscopy and scanning electron microscopy (SEM) with operating voltage of 20 keV (Supra 55, Zeiss, Oberkochen, Germany), equipped with energy dispersive spectrometry (EDS). The chemical compositions of different areas of the specimen are determined by electron probe microanalyzer (EPMA) (JXA-8100, JEOL, Tokyo, Japan). The non-isothermal oxidation experiments were also carried out using thermogravimetric (TGA) (SDT Q600, NSK, Tokyo, Japan) with accuracy of 0.01 mg for the comparison. The specimens were heated from room temperature to 1300 °C at the heating rate of 10 °C/min, which were under a flowing gas mixture of nitrogen (80 mL/min) and oxygen (20 mL/min) during heating.

## 3. Results

### 3.1. Non-Isothermal Oxidation

**Figure 2:**
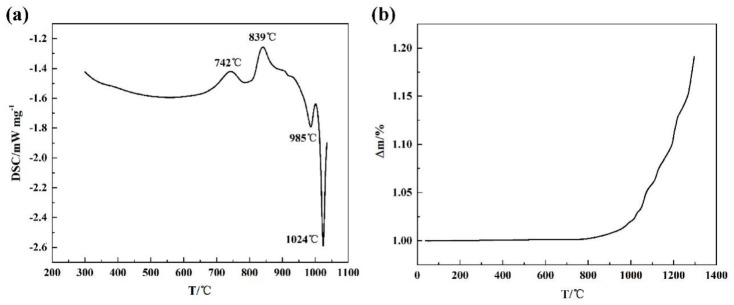
Differential scanning calorimetry (DSC) (**a**) and thermo gravimetric analysis (TGA) (**b**) of Ti14 alloy.

The non-isothermal oxidation results of Ti14 alloy containing DSC (differential scanning calorimetry) and TGA curves are shown in Figure 2. The thermograms in Figure 2a show two exothermic peaks near 740 °C and 840 °C, respectively. The first peak is probably attributed to the clustering of atoms and formation of Guinier–Preston zones. The second peak may be due to the precipitation of Ti_2_Cu as the final phase. Two endothermic peaks at higher temperature is also visible near 985 °C and 1024 °C. The peak at 985 °C corresponds to the melting point of Ti_2_Cu phase and second endothermic peak at 1024 °C is due to α/β phase transformation. The TGA carve of Ti14 from room temperature to 1300 °C is plotted in Figure 2b. The mass gains of the sample do not change at lower temperatures, however, the oxidation process is severely accelerated at 970 °C as reflected by the variation of the mass gains.

### 3.2. Combustion Characteristics

The combustion process is shown in Figure 1b–d, three stages can be distinguished from the PIC tests observation of the Ti14 alloy combustion process, i.e., thermo oxidation, ignition, and flame expansion. The combustion process of Ti14 is similar to that of Ti-Cr-V alloys which were described detailed in previous study [22]. However, some unique phenomena of Ti14 are observed during the combustion different from Ti-Cr-V alloys, such as several sparks of droplets splashing into the environment for Ti14 alloy. This phenomenon may be caused by the different solid solubilities of oxygen in different phases, formed during the combustion process of titanium alloys.

**Figure 3:**
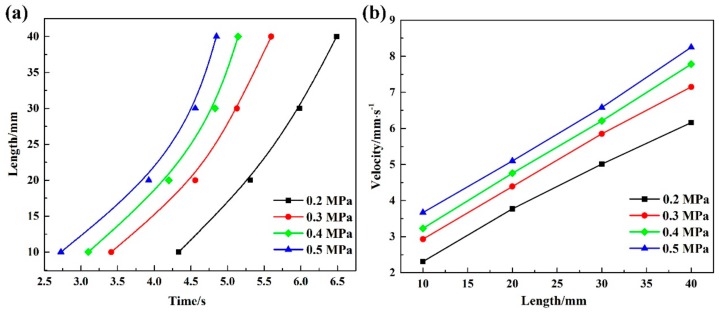
Burning length–time relationship (**a**) and burning velocity of different length (**b**).

The relationship between burning length and time is listed in Figure 3a. It can be seen from the picture that burning length has a parabola relation with the burning time at the same oxygen pressure. Moreover, it can be suggested by the variation of the slopes of length–time curves representing the burning velocity, the burning velocity is not a constant but changes with the burning time. The burning velocity at different pressures is shown in Figure 3b. As can be seen from the chart, similar changing trends of burning velocity are found at different oxygen pressures and the velocity is found increasing at the higher oxygen pressures. It is worth noting that the burning velocity increases with longer burning time at the same oxygen pressure, suggesting the combustion of Ti14 alloys is a self-accelerate reaction. The burning velocity of Ti14 corresponding to the 10 mm in length is 2.31 mm/s, then increased to 6.16 mm/s corresponding to the length of 40 mm at the oxygen pressure of 0.2 MPa. The variation of burning velocity at the same oxygen pressure may be attributed to the high combustion heat of titanium alloys. A huge amount of combustion heat is generated when the sample ignites, as the combustion process continues the heat accumulates gradually and eventually leads to accelerating velocity.

### 3.3. Microstructure and Composition Distributions of the Combustion Areas

**Figure 4:**
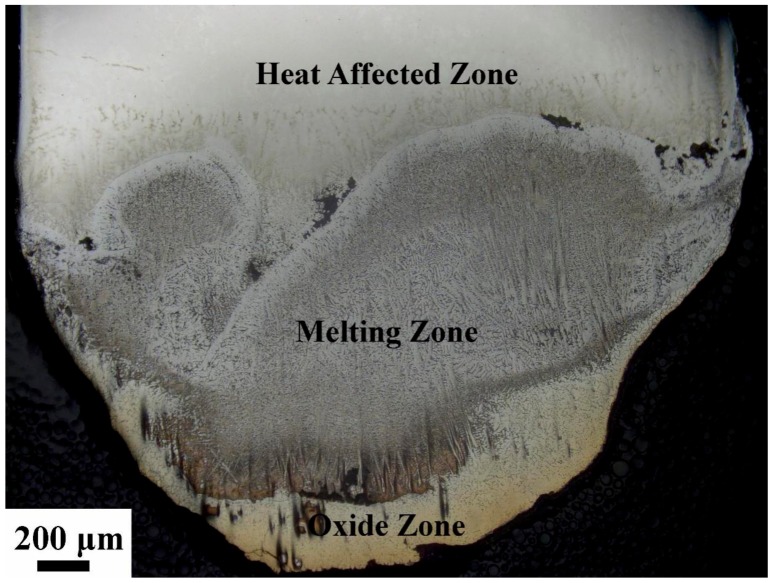
Overall morphology of Ti14 alloy, containing oxide zone, melting zone, and heat affected zone.

The combustion behavior of titanium alloys is different from that of copper alloys [23]. Once it is ignited, the sample of titanium alloys burn completely due to its high combustion heat. There are three different morphology of Ti14 alloy after combustion, those are oxide zone, melting zone, and heat affected zone, as shown in Figure 4. The oxide zone is the burning product which located in the outermost layer of the sample. The melting zone is formed as a result of intensive temperature influence, inhibited by the oxide zone which has already formed. In the heat affected zone, which is located farthest from the heat source, the microstructure is also affected by the heat from the melting zone, leading to the coarsening of grains but no phase transformations.

**Figure 5:**
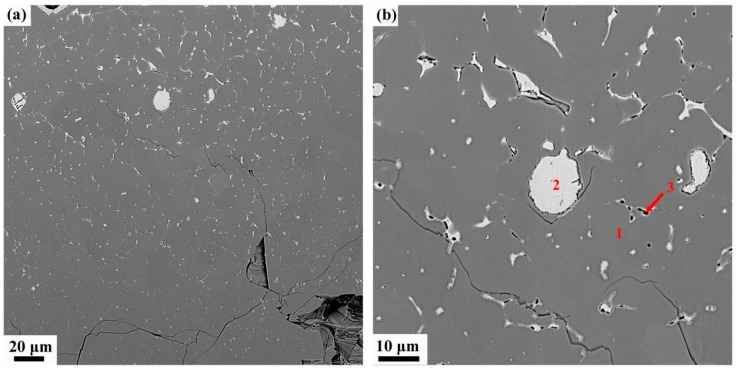
Microstructures of the oxide zone of Ti14 alloy, (**a**) overall morphology of oxide zone, (**b**) typical oxide zone, and 1, 2 and 3 marks are three different phases and their compositions are shown in Table 2.

**Figure 6:**
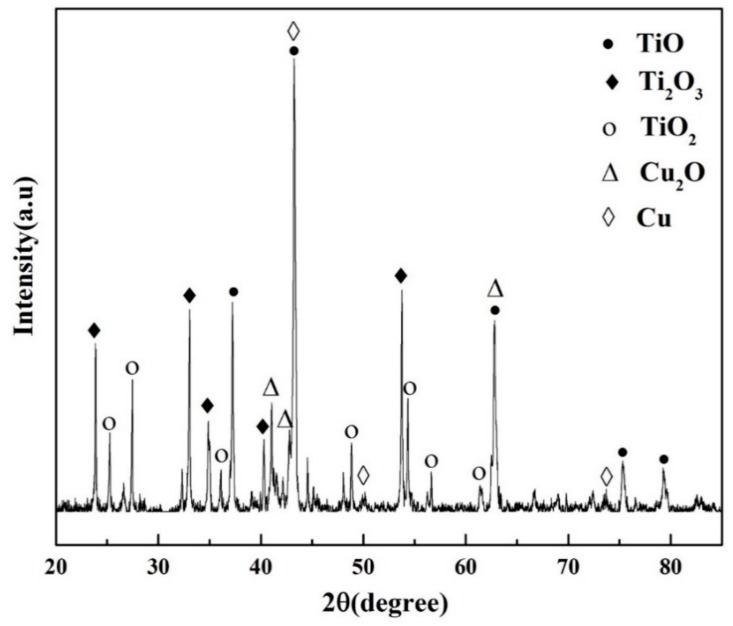
XRD analysis results of oxide zone after combustion.

In the oxide zone, the burning product shows a lot of cracks which benefits the oxygen transportation and against the burn resistance, as shown in Figure 5a. Those cracks may be attribute to the thermal stress in the cooling process. It can be seen from Figure 5b, there are three different phases can be distinguished in oxide zone, i.e., the gray matrix phase (phase 1), the white phase (phase 2) and the black particle phase (phase 3). The gray matrix is probably composed of Ti and O and appears to be titanium oxide based on the chemical composition by EPMA listed in Table 2. The composition of the phase 2 mainly consisting of Cu, and a little Ti and Al, may be phase 2 not burning completely. Phase 3 is found surrounded by phase 2, containing more amount of Ti. X-ray diffraction is conducted to investigate the phase formation of oxide zone, as shown in Figure 6. According to the XRD, the phase 1 may be made up of TiO, Ti_2_O_3_ and TiO_2_ and the phase 2 is a mixture of Cu and Cu_2_O. The XRD does not reflect the phase 3 may be because of the little content of it.

**Figure 7:**
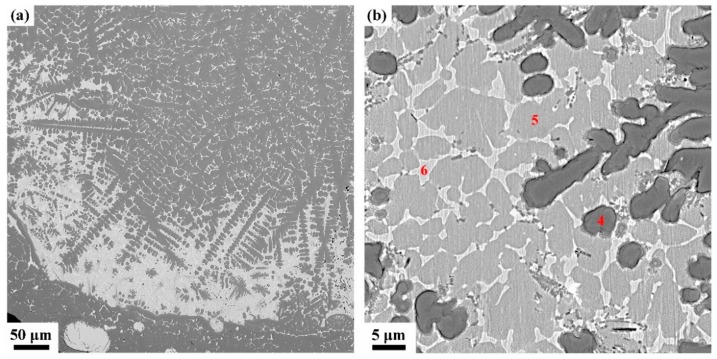
Typical microstructures of the melting zone of Ti14 alloy, (**a**) overall morphology of melting zone, (**b**) typical melting zone, and 4, 5, and 6 marks are three different phases and their compositions are shown in Table 3.

**Figure 8:**
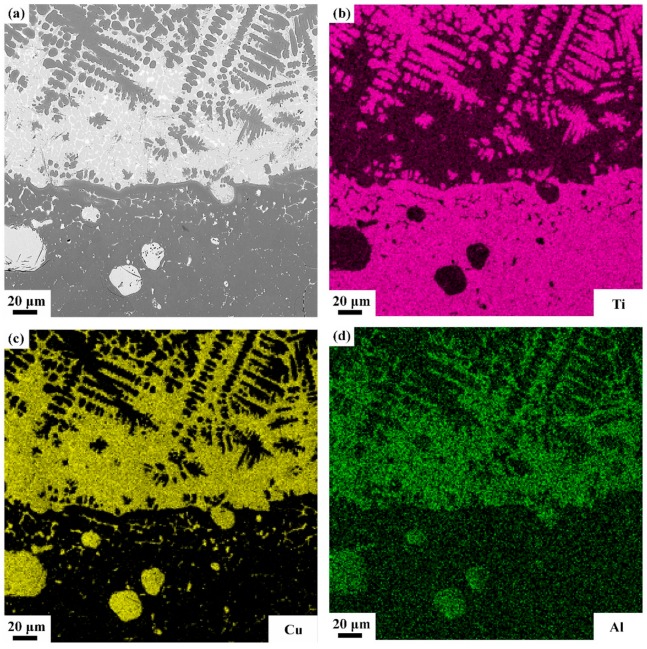
SEM photograph of typical microstructure of oxide zone and melting zone (**a**), and the corresponding mapping-scan of EDS analysis, containing (**b**), (**c**), and (**d**) for Ti, Cu, and Al atomic distributions, respectively.

The detailed microstructures of melting zone are shown in Figure 7, some pores and three different microstructures are found, i.e, the black dendrite structure (phase 4), the gray cellular structure (phase 5) and the white intergranular structure (phase 6). In the cooling process, the solubility of oxygen in liquid alloy is reduced and the O atoms desolated from the solid solution to form the O_2_ molecules. The cooling process is so quick that some O_2_ molecules do not have enough time to escape and form those pores. Phase 4 is possibly sub-oxides of titanium according to the elements content listed in Table 3. Phase 5 contains more Cu but less Ti and O than phase 4, and the intergranular structure (phase 6) which extreme enrichment of Cu element. There is an obvious interface between oxide zone and melting zone and in the interface the Cu content is pretty high according to the EDS mapping shown in Figure 8.

**Figure 9:**
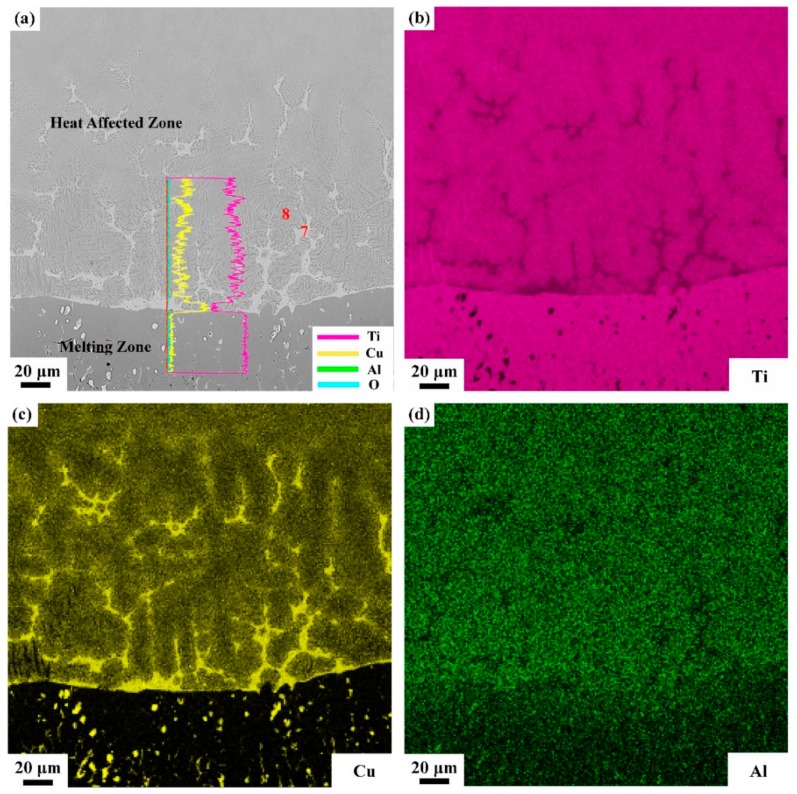
SEM photograph of typical microstructure of heat affected zone and melting zone and the corresponding line-scan of EDS analysis (**a**), the mapping-scan containing (**b**), (**c**), and (**d**) for Ti, Cu, and Al atomic distributions, respectively.

In the heat affected zone, grains became coarser than matrix and some white net-like structures (phase 7) are found. The Cu content in phase 7 is about twice as Ti. The phase 8 is close to the nominal composition with a little of O, as list in Table 4. A clear interface is also found between the melting zone and heat affected zone, and the EDS line scan and mapping scan are performed cross the interface as shown in Figure 9. It can be seen from Figure 9 that, in the interface, the Cu content increases sharply which means the Cu element is enriched in the interface between melting zone and heat affected zone.

## 4. Discussion

According to the TGA results, Ti14 alloy oxidizes violently when the resistance wire heats the alloy to 970 °C and releases a tremendous amount of heat. Then the titanium alloy is ignited when the heat accumulates up to the ignition point. According to the reference [24], the flame temperature of titanium alloy is about 2930 °C which is above its melting point. Therefore, the titanium alloy begins to melt into liquid phase during combustion process. The quantity of dissoluble oxygen in liquid alloy is found much higher than that in solid alloy which causes the more intensive response. The combustion reaction continues until it burns completely. The equilibrium microstructures of Ti14 alloy consist of Ti_2_Cu and α phases. Ti_2_Cu phase shows the low melting point about 985 °C and it melts in thermal oxidation process before ignition which absorbs a lot of heat to the hinder the ignition process. During the combustion, the temperature of alloy is heated up to 1740 °C and the peritectic reaction: L + α → β would probably occur in the alloy with 1–13.5 at % oxygen according to the Ti-O phase diagram. The maximum solid solubility of O in β phase is 6 at %, so the supersaturated oxygen atoms will desolate and assemble into oxygen molecules. Finally, those oxygen molecules escape from the melted alloy and cause the sparks splashing into the environment. As the combustion progresses, more oxygen takes part in the reaction and the second peritectic reaction: L + α → TiO occurs when the oxygen content exceeds 35 at % at 1770 °C or higher temperatures. A part of TiO continue to react with O and to form the Ti_2_O_3_ and TiO_2_. This analysis is consistent with the XRD and EPMA results.

Standard formation of free energy (∆G^θ^) for metallic oxide reflects the stability of oxide and the value of ∆G^θ^ can be obtained from the oxygen potential diagram. It is well known that the affinity between the metal and oxygen is stronger and the corresponding oxide is more stable if the value of ∆G^θ^ is more negative. It can be seen from the oxygen potential diagram, the ∆G^θ^ of Ti is much more negative than that of Cu which means the affinity between Ti and O is stronger and Ti reacts with O preferentially during combustion. Therefore, the metallic oxides generated by the reactions between Ti and O are found in the outermost region of the sample to form the oxide zone. Because of the preferential reaction of Ti and O, the Cu element is consequently enriched in the interface between the oxide zone and melting zone as shown in Figure 8. Those oxygen atoms are difficult to diffuse through the Cu enrichment zone due to the low dissolved oxygen of Cu element and it hinder the oxygen diffusing to the melting zone. In the heat affected zone, the microstructures of Ti14 matrix is found coarsened attributed to the combustion heat. The Ti_2_Cu phase shows the low melting point which is about 985 °C. During the combustion, the Ti_2_Cu phase melts first than matrix and forms the white net-like structures in heat affected zone as shown in Figure 9. The EPMA results show that the phase 7 is Ti_2_Cu phase which is agree with the analysis. There is another Cu enrichment zone in the interface between melting zone and heat affected zone caused by the Ti_2_Cu phase preferential melts. It can prevent the O diffusing to the matrix further and shows good burn-resistant effect to some extent.

The combustion behaviors of Ti14 is similar to that of Ti-Cr-V system alloys which was reported in the previous study of the author [22]. They both have an element enriched zone between oxide zone and melting zone which enriched with Cr and V for Ti-Cr-V alloys and Cu for Ti14 alloys. The element enriched zone shows the low combustion heat than the matrix since the combustion heat of Cr, V, and Cu (2608 cal/g, 3637 cal/g, and 317 cal/g, respectively) is lower than that of Ti (4717 cal/g). Less heat is released when the flame extends to the element enriched zone and the burning velocity is slowed down. Moreover, the Cu element is considered more powerful than Cr and V in the terms of burn resistant due to its lesser combustion heat and lower solubility of oxygen. However, there are some differences of combustion behavior between Ti-Cr-V and Ti14 alloys. The Ti-Cr-V alloys show the single β phase structure and Ti14 alloy shows the double phases structure which contains α and Ti_2_Cu phases at service temperatures. The solid–liquid interface moves forward through the grain boundary for Ti-Cr-V alloys as reported in [22], whereas Ti14 alloys in a different way. Ti_2_Cu phase melts firstly into liquid phase due to the low melting point before the ignition, which causes the hindrance of ignition since the heat transfer is promoted by the liquid phase. The peritectic reaction (L + α → TiO) as mentioned above, is considered as the key factor to control the burning velocity, since this reaction may occur in the solid–liquid interface leading to the solid–liquid interface move forward. Therefore, the different combustion behavior of Ti14 and Ti-Cr-V alloys is basically controlled by the characteristics of phase structures and chemical reactions.

## 5. Conclusions

This paper studied the combustion behavior of Ti14 alloy by the PIC tests at different oxygen pressures to reveal the influence of element enrichment and phase structure on combustion mechanisms. The following conclusions can be drawn:

(1) The burning velocity of Ti14 alloy is found increasing at the higher oxygen pressures and increasing with longer burning time at the same pressure instead of a constant, suggesting that the combustion of Ti14 alloy is a self-accelerating reaction.
(2) The combustion reaction area of Ti14 alloy is found containing three different zones, i.e., oxide zone, melting zone and heat affected zone. The Cu atoms are found enriched in two zones—i.e., the heat affected zone and melting zone during the combustion process—which can prevent the diffusion process of oxygen atoms.
(3) The combustion behavior is related to the phase structure. The occurrence of peritectic reaction: L + α → TiO is deduced in the solid–liquid interface during the combustion process by the analysis of the chemical composition and phase contents of the reaction area, regarded as the key factor that decides the moving velocity of the solid–liquid interface.

## Figures and Tables

**Table 1:** Chemical composition of the samples

Element	Ti	Al	Cu	Si
at %	86.35	2.25	10.72	0.68
wt %	84.46	1.24	13.91	0.39

**Table 2:** Chemical compositions of different phases in the oxide zone

Region	Composition				
	Ti	Cu	Al	Si	O
1 (at %)	47.82	0.04	0.28	-	51.86
1 (wt %)	73.16	0.08	0.28	-	26.51
2 (at %)	6.10	88.87	4.06	0.20	0.77
2 (wt %)	4.81	93.08	1.81	0.17	0.20
3 (at %)	85.36	2.13	2.38	0.25	9.88
3 (wt %)	91.81	3.04	1.44	0.16	3.55

**Table 3:** Chemical compositions of different phases in the melting zone

Region	Composition				
	Ti	Cu	Al	Si	O
4 (at %)	70.26	0.52	0.30	0.04	28.88
4 (wt %)	86.96	0.85	0.21	0.03	11.94
5 (at %)	64.50	29.20	3.20	0.51	2.59
5 (wt %)	60.72	36.48	1.70	0.28	0.81
6 (at %)	30.04	54.68	13.42	0.13	1.73
6 (wt %)	27.11	65.48	6.82	0.07	0.52

**Table 4:** Chemical compositions of different phases in heat affected zone.

Region	Composition				
	Ti	Cu	Al	Si	O
7 (at %)	66.67	28.88	1.28	0.54	2.63
7 (wt %)	62.36	35.85	0.67	0.30	0.82
8 (at %)	82.29	9.25	2.11	0.40	5.95
8 (wt %)	83.99	12.53	1.21	0.24	2.03
